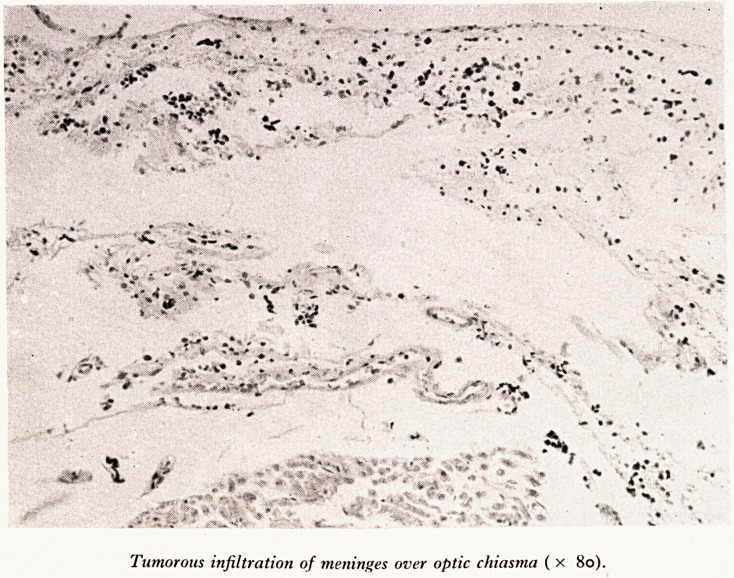# Malignant Ependymoma Masquerading as Tuberculous Meningitis

**Published:** 1962-01

**Authors:** T. F. Hewer


### MALIGNANT EPENDYMOMA MASQUERADING AS TUBERCULOUS MENINGITIS

*A Clinico-Pathological Conference of the University of Bristol held on the 24th October*, 1961

CHAIRMAN: PROFESSOR T. F. HEWER

### P.M. No. 7023

*Dr. Crow:* This is the case of a young boy of 15; his clinical history was tragically brief and should not take long to recount. The doctor from a public school in Somerset rang me up as Dr. Campbell's Registrar on the 17th May 1961 and said he had just seen a young boy at school who had a 10-day history of headaches, vomiting and seeing double. Did I think he ought to come into hospital? I said I thought he should and he was taken in that day. I saw the boy in the afternoon and he seemed perfectly healthy. He was cheerful and quite bright. He did not make much of his headaches. He said he had not had headaches in the past, but for the last 10 days he had been having throbbing frontal headaches. They had not been particularly severe, did not occur at night and were not there in the morning, but came on later in the day. On the 15th May 1961 he had vomited once in the morning and on the 17th he had vomited twice in the morning and felt a little dizzy. I asked him if he had diplopia and he said he had been wearing glasses recently, and over the last week, whenever he took them off in the classroom, he saw double for a few minutes, but it cleared up. I was in some doubt as to whether in fact this boy was really ill, but we admitted him and I arranged for him to have a lumbar puncture next morning. X-rays of the chest and skull were done and there was no abnormality. His blood count was entirely normal, as was his E.S.R. Lumbar puncture was done and on the afternoon of the day after his admission we got the report. The c.s.f. pressure was over 200 mm of water, but it had not been an easy lumbar puncture, as he was apprehensive, and we thought at the time that it was because he was not completely relaxed. The c.s.f. showed 32 cells/c.mm, almost all of them lymphocytes. The protein was 92 mgm per cent and, most significantly of all, the sugar content was 23 mgm per cent. Now this, I felt, was absolutely typical of tuberculous meningitis. Pleocytosis and rise of protein do not necessarily mean tuberculous meningitis; any type of lymphocytic meningitis might have given those results, but with a sugar as low as 23 mgm per cent, when the lower limit of normal is usually taken to be 50 mgm per cent, it seemed that this could only mean one thing, and that was tuberculous meningitis. The history fitted it, a short history of progressive headaches.

Now this boy was, as I say, perfectly clear mentally and we thought there should not be much difficulty here; he ought to respond to treatment because with modern methods of treatment patients who are conscious on admission to hospital have an extremely good prognosis. If they are already bad enough to be unconscious then the prognosis is not nearly so good. However we did not start him on treatment immediately because I thought it would be advisable to get another specimen of c.s.f., a large specimen which could be concentrated and which could be used for a search for tubercle bacilli and for a guinea-pig inoculation. With tuberculous meningitis you are condemning patients to an arduous and lengthy course of treatment and it is always comforting to have bacteriological proof. We had to wait until the next morning when we did a second lumbar puncture and then started him on treatment. The second lumbar puncture showed virtually the same finding as the first, the cell count was slightly higher, 43 per c.mm, protein the same. The sugar was down to 19 per cent. He was started on intra-muscular streptomycin, oral P.A.S. and I.N.H. We did not give him

PLATE VII

*Healing Ghon's focus in lung* (× 80).

PLATE VIII

*Ependymoma of IVth ventricle causing obstructive hydrocephalus* (× 0·7).

PLATE IX

*Ependymoma of IVth ventricle: herniation of cerebellar tonsils* (× 1·2).

PLATE X

*Rosette formation in ependymoma* (× 150).

PLATE XI

*Malignant spread of ependymoma to meninges over cerebellum* (× 80).

PLATE XII

*Subependymal spread of tumour in base of IIIrd ventricle* (× 80).

PLATE XIII

*Tumorous infiltration of meninges over optic chiasma* (× 80).

any intrathecal treatment at this point. There have been publications of tuberculous meningitis being cured without the use of intrathecal streptomycin. It is customary however to use intrathecal streptomycin for the first week or 10 days and this we intended to do, but I was still most anxious to get bacteriological proof, and once you give intrathecal streptomycin you greatly reduce the possibility of achieving bacteriological proof. Furthermore you get a fairly brisk reaction from the meninges to the streptomycin, so that the cell count and the protein count normally rise. We therefore held up on this occasion from giving intrathecal streptomycin. For the first two days his condition did not change. He vomited once or twice but he did not complain much of headache. The he went downhill with the most alarming rapidity. He developed very severe headaches, becoming a little drowsy and confused, then restless and noisy, (I should have said he was apyrexial), and I certainly became extremely alarmed at this, and on his 4th day we repeated the lumbar puncture and on this occasion gave him intrathecal streptomycin as well. The third specimen of c.s.f. showed a pressure of 230 mm, the cell count was now 167/c mm, protein was actually slightly reduced at 64, sugar oddly enough had gone up to 45 mgm per cent. By the fourth day he was vomiting quite a lot and we stopped feeding him by mouth and gave him an intravenous tranfusion of glucose and saline, we also gave him some Avomine intramuscularly (an anti-histamine preparation which is a fairly potent anti-emetic) and he actually did stop vomiting. We started him too on steroids. Now the use of steroids in tuberculous meningitis is still debatable, but many people who have experience of this condition think that, if a patient is toxic and ill in the early stages of the meningitis, the use of steroids is helpful. I do not think anyone has produced any statistical proof but the clinical impression is very strong that it reduces the severity of the illness while the treatment is getting going. So we started him on steroids. That very evening he was found dead in bed.

He had seemed a little better early in the evening. He had stopped vomiting, he was quiet, conscious but very drowsy. His death was quite unexpected. We assumed that he had vomited, in a drowsy stuporose state, inhaled his vomit and died in that way. We were extremely upset, of course, and we looked to the pathologists to give us an answer we hoped would relieve our uneasy consciences.

*Question:* Were there no neurological signs?

*Dr. Crow:* None of any kind. I can't say what happened to the fundi I am afraid. Certainly initially the fundi were absolutely normal. We dilated the pupils on admission and hunted for retinal tubercles because one of the ways in which you can be certain of tuberculous meningitis in the early stages before you can get bacteriological proof is by finding retinal tubercles. They can usually be found in a high percentage of cases by diligent search and some people have said that the incidence of retinal tubercles found is an indication of the diligence of the search. However we failed to find them. I myself did not look at the fundi again when he had started going downhill. I think they might have shown some papilloedema but this would not have altered our treatment; I should have done exactly the same.

*Dr. Coles:* I presume he had not had B.C.G.?

*Dr. Crow:* So far as we know he hadn't, but there was no previous history and no history of contacts.

*Professor Hewer:* Just a point of comment on retinal tubercles: their incidence is partly a function of the diligence of the search, but I find it very hard to believe that they are necessarily an indication of tuberculous meningitis. I am sure you find them in miliary tuberculosis without meningitis.

*Dr. Crow:* Oh yes, I think so. If you have a case of meningitis and you see tubercles in the fundi, then you can say this is tuberculous meningitis. It does not follow the other way round.

*Professor Hewer:* The point, a slightly academic one perhaps, is that tuberculous meningitis means tubercle bacilli in the subarachnoid space, not in the brain, and what you are seeing in the retina is a blood-borne tubercle. The retina happens to be the only place you can see the tubercle. You can have miliary blood-borne tuberculosis without the meningitis but very often the two are associated.

*Question:* Have you any record of his blood pressure in the last 12 hours?

*Dr. Crow:* No, I'm afraid not.

*Dr. Sanerkin:* Can you tell us about the Mantoux reaction?

*Dr. Crow:* I am sorry I can't give you the result. The injection was made on his first day of admission to hospital but I cannot remember what the response was, and it is not recorded in the notes.

*Dr. Lloyd:* It would probably have been positive.

*Dr. Coles:* It might equally have been negative. We recently had a gentleman whom Professor Milnes Walker would remember who had tuberculous peritonitis and on repeated Mantoux tests showed a negative result.

*Dr. Crow:* There was one difficult point about getting a post-mortem examination. This boy's parents were abroad, he was under the care of an aunt who at the time of his death was not available. We had seen her, but had been relatively reassuring and she had gone off to somewhere, where we couldn't contact her. When this patient died I knew that something had gone wrong; I felt he should not have died and as his death weighed heavily on my conscience and on the consciences of the ward staff I thought it best to report it to the coroner.

*Professor Hewer:* You could not get in touch with the relatives at all anyway.

*Dr. Crow:* No, sir. His guardian had left the address we knew and we did not contact her for some days.

*Professor Hewer:* What did the coroner say?

*Dr. Crow:* I cannot say, sir.

*Professor Milnes Walker:* The only alternative to referring the case to the coroner was to sign it as tuberculous meningitis.

*Dr. Crow:* Exactly. But his course was so atypical that it looked as though he had died as the result of some fault in treatment or in care and because his parents were abroad I felt we must get the record straight.

*Dr. Lloyd:* Is it not a fact that in the absence of the nearest relative the Board of Governors of the hospital are the custodian of the body, and can give permission?

*Dr. Hunt:* There is a new Act (Human Tissue Act, 1961) which is now in operation which gives details of who is in fact the custodian of the body. Up until that time there was no property in a dead body and nobody could be the guardian of something which did not exist in law. Now that it does apparently exist in law you must have permission from the guardian before performing a post-mortem. Before the Act permission for post-mortem was not strictly necessary but we always have asked for it as a matter of courtesy. Now, if you cannot contact the lawful custodian of the corpse, the governing body of a hospital is empowered to give permission. But this Act only came into force about a fortnight ago and obviously did not apply in this case.

*Dr. Lloyd* then gave the post mortem findings: This was a most misleading case and you will be delighted to hear that the pathologist fell into the same trap as did the clinicians.

He was a well nourished young man with extreme cyanosis, more particularly of the head and neck. The heart showed dilatation of the chambers, more on the right than than on the left; it was otherwise normal. He had a little bit of atheromatous streaking of the aorta, young though he was, but there was no fibrosis. In the pharynx there were scars of old tonsillectomy. His lungs showed acid digestion and oedema, more on the right side than on the left; that was because of the inhalation of vomitus, exactly as Dr. Crow had prophesied. I thought this case was likely to be one of tuberculous meningitis and I searched therefore very carefully through these lungs for a primary tuberculous focus. I found one (3 mm in diameter) in the upper part of the right lower lobe; it was rather calcified and had a fibrous capsule and was not particularly active. Moreover I could not find any tuberculous lesions in the corresponding lymph nodes and I ought to have been a little bit more cautious therefore, because it is a little unlikely that you would get meningeal spread from a primary focus in the lung which is healing, as you will see this one is. (Plate VII.) In this Ghon's focus there is a capsule in the periphery of which there are still some histiocytes and fibroblasts which are mildly active, but the rest of the capsule is composed of hyaline connective tissue. There is a little calcified necrotic mass in the centre. There were no lesions in the spleen or liver. There were some acute erosions in the stomach and altered blood could be found in that viscus. There was no haemorrhage in either adrenal gland. The brain was the only other organ which showed any pathological abnormality. Here I found that there was slight flattening of the convolutions; this was due to the internal hydrocephalus. I examined the external surface very carefully to see if I could find some tubercles and I found some little bodies of the right size which would do very well for them. The outer surface of the cerebellum showed some cloudiness over little swellings which were distorting the pattern of the folia. Besides these I recorded observing about six of these little bodies on the surfaces of the parietal lobes and I also noticed that there was a cloudiness of the meninges in the interpeduncular region and around the optic chiasma, which is a very characteristic finding in tuberculous meningitis. So I thought that that was probably the correct diagnosis, and I took the brain and fixed it whole, so that I might cut it up in a few days and look for the Rich focus. The Rich focus, as you will remember, is the focus of tuberculous dissemination which has arrived in the brain or meninges by the haematogenous route and from which the tuberculous meningitis eventually arises by spread through the meninges. So I cut the brain into slices and found this (Plate VIII.) "That" said I, "is *not* a Rich focus, that is a tumour." The tumour measured about 1·5 cm in diameter and was situated in the fourth ventricle, causing obstruction to the passage of the cerebro-spinal fluid, dilatation of the Sylvian aqueduct and of the third ventricle and of the lateral ventricles. It was also causing herniation of the cerebellar tonsils (Plate IX) so that there was some blockage of the foramen magnum. All of these things contributed to the acute intracranial tension which was partly the cause of his death; it was the cause of his vomiting, and the acid digestion of his lungs was a contributory cause of death.

Microscopical examination of this tumour (Plate X) showed it to be an ependymoma. The rosette formation of slightly elongated cells is characteristic. I stained the tumour with phosphotungstic acid haematoxylin to see whether I could find any blepharoplasts, but I was unsuccessful.

The tumour was not confined to the fourth ventricle, it was spreading through the cerebellar substance onto the meninges (Plate XI), and this was the reason why he had the little diffuse and focal tubercles on the surface of the brain which I saw at post mortem. The tumour had also spread in the subependymal region throughout the whole of the ventricular system (Plate XII). The next picture (Plate XIII) shows the cause of the thickening and cloudiness of the meninges in the basal region where I had noticed it at post mortem. It is due to infiltration of these meninges by tumour.

The first thing which upset my diagnosis of tuberculous meningitis in this case was the failure to find tubercle bacilli in the tubercles from the surface of the brain. I got one of them and crushed it between slides and got Dr. Mitchell to stain it by Ziehl-Neelsen's method. He had a hunt for tubercle bacilli but was not able to find any and I later hunted for them myself, long and earnestly, and was equally unsuccessful. Now if you do that in a genuine case of tuberculous meningitis you are almost certain to find tubercle bacilli in those particular lesions.

This is in fact a primary tumour of the fourth ventricle, an ependymoma, which usually is a benign tumour, but which in exceptional circumstances like the present may be malignant and spreads in this fashion through the meninges and also in the subependymal parts of the brain. The reason why he got these queer c.s.f. findings, I have no doubt, is due to the fact that he had malignant infiltration in his meninges.

*Professor Hewer:* Obviously the most exciting and important thing here is the misleading character of the c.s.f. Here is a case which had all the criteria of tuberculous meningitis except for the actual organism and it is very disturbing from the point of view of clinical pathology.

*Dr. Crow:* This condition is recognized and called meningitis carcinomatosa. We have in fact had one or two cases in hospital in the last year or two. It certainly never crossed my mind to doubt the diagnosis seriously, but I did find afterwards that my junior staff had been discussing the possibility behind my back! I do not really see how one could arrive at a correct diagnosis in this case. In the cases of meningitis carcinomatosa that we have had the diagnosis has been rather easier because there has been evidence of a primary tumour elsewhere. In one case there was a primary carcinoma of the bronchus and when a meningitis was discovered and the cells from the c.s.f. were examined some were thought to be malignant and this proved to be correct. But in a case like the present one, arising out of the blue with such a short history, which was a history typical of tuberculous meningitis, the difficulty is great. Next time I suppose I will consider the possibility, but that will make a diagnosis of tuberculous meningitis even more difficult. However the treatment will still have to be that for tuberculous meningitis. One of the characteristics of meningitis carcinomatosa is a low level of sugar in the c.s.f. Quite how that arises I am afraid I do not know. In one of the previous cases, I remember, the c.s.f. sugar was down below 50 mgm per cent.

*Dr. Coles:* Yes, about 30, I think.

*Question:* Do you know what the c.s.f. chloride was?

*Dr. Crow:* We did not do it. In fact we have stopped doing the c.s.f. chlorides altogether. It is a labour for the laboratory and we pay no attention to it anyway.

*Question:* What was the time interval between the final lumbar puncture and the patient's death?

*Dr. Crow:* That is a very good question. The final lumbar puncture must have been performed sometime between 9 and 10 in the morning, and he died at 9.45 p.m., so that the interval was about 12 hours. The question is pertinent because his raised intracranial tension was due to a posterior fossa lesion, not as we thought to a generalized meningitis. In a meningitis the flow of c.s.f. is normal and there is virtually no risk in doing a lumbar puncture, in fact it may be a therapeutic procedure to relieve the c.s.f. pressure because it relieves the patient's symptoms. But if the rising pressure is due to a local space-occupying lesion which is blocking the c.s.f. outlet from the brain then of course to reduce the pressure by lumbar puncture is asking for trouble because the pressure above plugs the cerebellum and the brain stem down into the foramen magnum; or alternatively the brain may get pushed through the orifice of the tentorium cerebelli. I think it is quite likely that the final lumbar puncture precipitated a certain amount of increased intracranial tension.

*Professor Hewer:* In view of the diagnostic difficulties in this case I have asked Mr. Alexander to come and give us his comments.

*Mr. Alexander:* We know now that this is a case of fourth ventricle tumour and, looking back on the story I wonder whether the patient was not too ill for the small number of cells found in the c.s.f. A patient with tuberculous meningitis, as ill as this one became, might have been expected to have accumulated hundreds or a few thousands of cells. The restlessness sounds like acute hydrocephalus. I looked at Bailey and Buchanan on "Intracranial Tumours of Childhood"; they give accounts of seven ependymomas, all in the fourth ventricle. There were no lumbar punctures except in one that had thirteen cells (similar finding in this case) but there was no mention of the sugar content. That patient died soon after the lumbar puncture, and there was an autopsy, but no operation. I think the case under discussion was clinically most difficult, with the low sugar. This boy had in the long run no chance, even if his tumour had been taken out, because he would have had seedlings ultimately in the spinal canal, the place where malignant ependymomas deposit. He also had some in the ependyma elsewhere, so that he would have needed to have been treated by radiation of skull and of the spinal column to have given him a palliative chance. I think it is a consoling thought, that he was doomed anyway with this malignant growth.

*Professor Milnes Walker:* May I ask Mr. Alexander how frequent are these fourth ventricle tumours compared with the ones in the lateral ventricles?

*Mr. Alexander:* Lateral ventricle tumours of this sort are curiously uncommon. In the reported cases they are mostly in the fourth ventricle. The ependymoblastoma, however, which is of a pinkish colour compared with this, occurs in the hemispheres. The other thing I got out of Bailey and Buchanan is the distressing frequency with which the subjects were found dead in bed, having tolerated the operation well, although I suspect that in those days personnel were not drilled to keep the patient semi-prone after operation; if that is done patients cannot aspirate vomitus.

*Professor Hewer:* Is that done for all brain tumour operations?

*Mr. Alexander:* It is in most. Tracheostomy of course adds a safeguard, if the patient is unconscious.

*Dr. Spence:* Could I ask whether there is a particular age or sex incidence in this condition, in view of the difficulty of diagnosis?

*Mr. Alexander:* It occurs in children of either sex, it may occur up to the age of 60. But in general it is a child's tumour, in the fourth ventricle, and it is not common.

*Professor Hewer:* Mr. Alexander referred to the low cell count which I take it was the most inportant feature against tuberculous meningitis. On the other hand if tuberculous meningitis is getting better under treatment the cell count falls, the protein does not.

*Mr. Alexander:* Of course I never have seen cases of early tuberculous meningitis with fluids containing as little as 20 to 30 cells, but some intracranial cases of tuberculoma, which abounded in Scotland in the old days, were found to have cell counts of this order.

*Dr. Crow* (in answer to a question): I think in fact that there would probably have been papilloedema before his death. There certainly wasn't when he came into hospital. All I say is that raised intracranial pressure does not invariably give rise to papilloedema, or probably it would be more correct to say that papilloedema may appear late in the presence of raised intracranial pressure. Mr. Alexander, I think, would agree?

*Mr. Alexander:* Yes, that is quite correct, there is no history amongst those I have read in the books like this one. This was a swift thing. The usual story is of vomiting for perhaps 2 months, with headaches, perhaps early, but then passing off as the sutures stretch in the very young. But the history in this case appears to be unique.

*Dr. Crow* (in answer to question)*:* Even in an ordinary meningitis the mechanism by which the c.s.f. sugar becomes reduced is not absolutely known. It is believed not to be because it is used up by the bacteria. In the present case I do not suppose it was because the malignant cells used up the sugar but I am afraid I just do not know.

*Professor Hewer:* Tumour cells in culture do use up sugar.

*Dr. Crow:* I think it might be that.

*Professor Hewer:* This was a growth of tumour, as you say a meningitis carcinomatosa.

6

*Dr. Crow:* I assumed, in my simplicity, that in ordinary meningitis bacteria, as they do on media, use up sugar, but I am told that that is not the case.

*Professor Hewer:* It is the polymorphonuclear leucocytes themselves that use up the sugar.

*Question:* Is it worth looking for malignant cells in cerebral malignancy in general?

*Answer:* Yes, surely.

*Dr. Lloyd:* I do not think they would have missed it here somehow, because the malignant cells were about 2 or 3 times the size of lymphocytes and I think they would have spotted that and gone on as they did in the previous case, when they did not know what they were.

*Mr. Alexander:* In some cases of cerebral tumour, particularly glioblastoma, "lymphocytes" are present in the c.s.f. as a result of the meningeal reaction.

*Question:* What about the mechanism of the raised lumbar puncture pressure?

*Mr. Alexnader:* In an obstructive hydrocephalus, where there is a cerebellar cone. After lumbar puncture this cone may plug the foramen magnum, thus interfering with the function of the medulla and causing sudden death.

*Professor Hewer:* It presumably interferes with the flow of c.s.f. over the cerebellum.

*Mr. Alexander:* Yes, the tonsils are crowded together and the foramen of Majendie is in that crowding, so that when the cerebellar tonsils get plugged into the foramen magnum down goes the lumbar c.s.f. pressure.

*Dr. Crow:* In a case of an obstructive hydrocephalus you do get a raised c.s.f. pressure in the lumbar region, I presume that is because the intra-cerebral pressure is enormously raised and that must be transmitted down to the lumbar theca until you actually do push the cork into the foramen magnum, so to speak.

*Professor Hewer:* In summary, then, this was a case of a malignant ependymoma involving the meninges, presenting with the symptoms and signs of a case of tuberculous meningitis. The diagnosis was extremely difficult and the outcome necessarily fatal. Most people here seem to agree that if they were to meet another such case they would give it the benefit of the doubt and treat it as though it were one of tuberculous meningitis.

[The previous case of meningeal carcimomatosis referred to in the above discussion was that reported in this *Journal* in 1960, Vol. 75, p. 105.—Ed.]

## Figures and Tables

**Figure f1:**
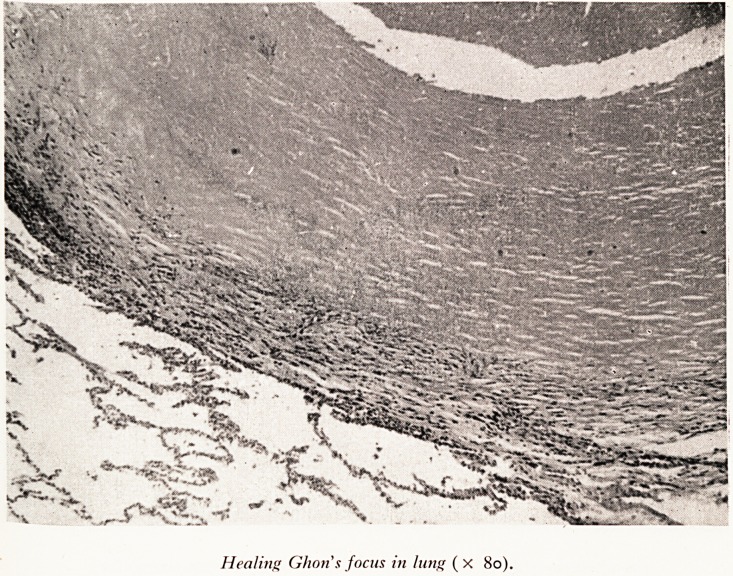


**Figure f2:**
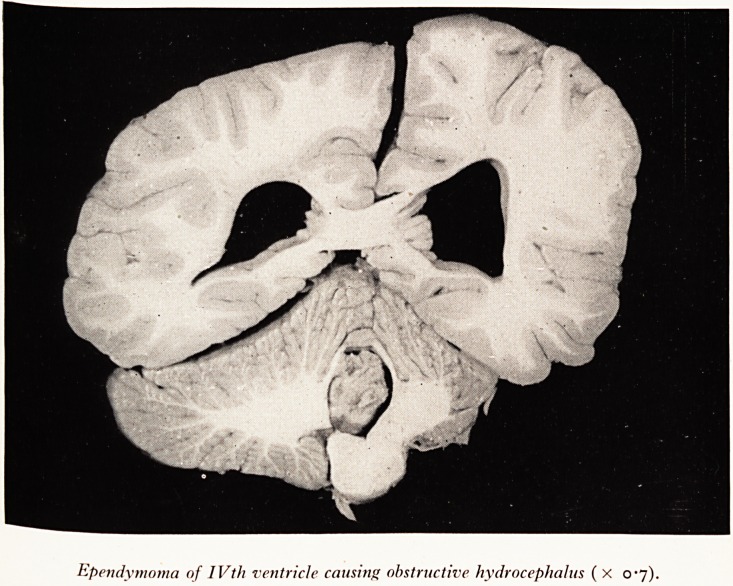


**Figure f3:**
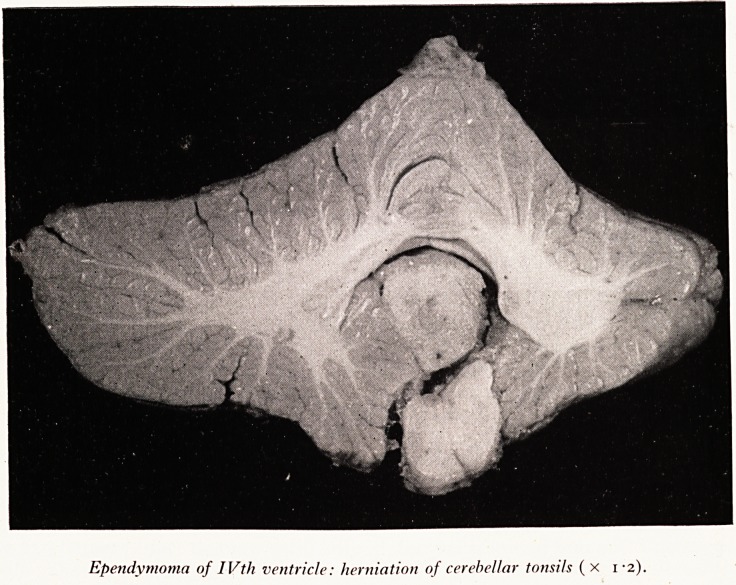


**Figure f4:**
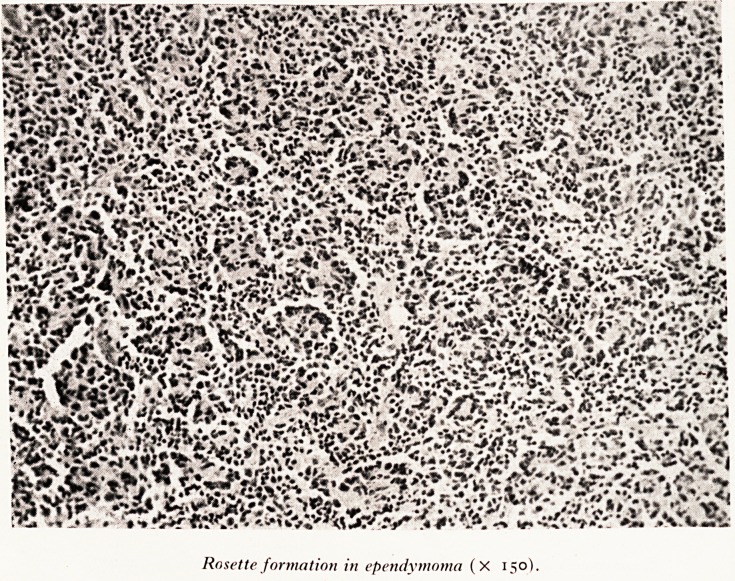


**Figure f5:**
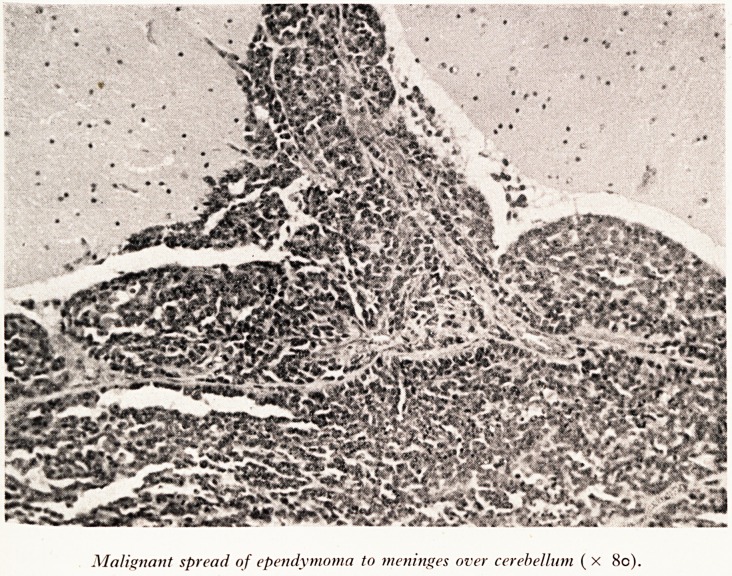


**Figure f6:**
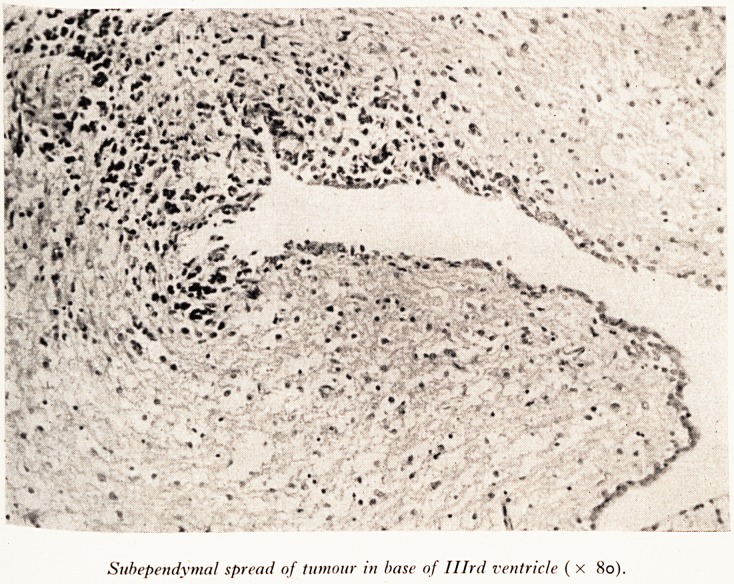


**Figure f7:**